# Cervical Health in Systemic Lupus Erythematosus

**DOI:** 10.1089/whr.2023.0023

**Published:** 2023-07-12

**Authors:** J. Patricia Dhar, Heather Walline, Gil Mor, Lamia Fathallah, Susanna Szpunar, Louis Saravolatz, Thomas Carey

**Affiliations:** ^1^ Department of Internal Medicine/Rheumatology, Wayne State University School of Medicine, Detroit, Michigan, USA.; ^2^ Rheumatology Fellowship Program, Department of Internal Medicine/Rheumatology, Ascension St. John Hospital, Detroit, Michigan, USA.; ^3^ Department of Otolaryngology-Head and Neck Surgery, University of Michigan Medical School, Ann Arbor, Michigan, USA.; ^4^ C.S. Mott Center for Human Growth and Development, Detroit, Michigan, USA.; ^5^ Department of Obstetrics and Gynecology, Wayne State University School of Medicine, Detroit, Michigan, USA.; ^6^ Department of Physiology, Wayne State University School of Medicine, Detroit, Michigan, USA.; ^7^ Department of Microbiology and Immunology, Wayne State University School of Medicine, Detroit, Michigan, USA.; ^8^ Ascension St. John Hospital, Detroit, Michigan, USA.; ^9^ Central Michigan University, Mount Pleasant, Michigan, USA.; ^10^ Biomedical Investigations and Research, Ascension St. John Hospital, Detroit, Michigan, USA.; ^11^ Department of Medicine, Wayne State University School of Medicine, Detroit, Michigan, USA.; ^12^ Department of Pharmacology, University of Michigan Medical School, Ann Arbor, Michigan, USA.

**Keywords:** SLE, lupus, HPV, cervical cancer, cervical dysplasia, gynecologic health

## Abstract

**Objective:**

A health disparity exists for African American (AA) women with systemic lupus erythematosus (SLE) who have increased prevalence of human papilloma virus (HPV) infection and cervical neoplasia. We used a self-sampling brush to obtain cervical cells to assess cytology, HPV infection, and vaginal cytokine production in AA women with SLE.

**Methods:**

Thirty AA women with SLE ages 18–50 years consented to participate. Clinical information was obtained by review of records and patient interviews, and surveys administered to assess cervical health history, knowledge of HPV, and satisfaction with the self-sampling brush. Vaginal samples were analyzed for cytology, HPV DNA and RNA, and vaginal cytokine RNA.

**Results:**

Our cohort (mean 36.9, ±9.4 years) had moderate/severe SLE and were on immunosuppressives. The majority had history of abnormal pap smears (63%) with prevalent risk factors for HPV infection: multiple sex partners (9.5 ± 7), not vaccinated for HPV (83.3%), smoking (26.7%), and not using condoms (73.3%). Most were aware of HPV causing cervical cancer (70%) but were unaware of other HPV-related diseases. Most preferred self-sampling over traditional pap smear (80%). Abnormal cytology was detected in 13.3%. HPV DNA was detected in 70%, with half showing multiple types, and all showing active infection (+RNA). HPV-infected samples demonstrated RNA expression of multiple cytokines with no specific/ consistent pattern.

**Conclusion:**

Our high-risk cohort lacked knowledge about HPV-related diseases and were not employing strategies to reduce their risk with vaccination and condoms. This study highlights the need for cervical health education, increased monitoring, and intervention in these high-risk women.

## Introduction

African American (AA) women bear significant disease burdens for both systemic lupus erythematosus (SLE) and cervical cancer with higher proportions for presentation of cervical cancer at later stages, as well as increased morbidity and mortality for both diseases.^1–5^ AA women with lupus encompass an underserved population residing in urban areas concentrated in settings with socioeconomic factors that result in lack of resources and poor social determinants of health. Consequently, this group of women has poor access to health care due to barriers in insurance, resources, education, and social support.^6^

Current guidelines by the American College of Obstetricians and Gynecologists (ACOG) and the United States Preventive Services Task Force (USPSTF) for cervical cancer screening are inadequate to monitor these high-risk women, and there are no specific recommendations for women with SLE such as exists for women with HIV. Pap smear testing requires an invasive examination by a trained health care professional in an office setting. Underserved populations with poor resources may have social and economic barriers that prevent compliance with this type of preventive health care.

Little is known about the biology and genetics of human papilloma virus (HPV) infection and related cervical disease in women with SLE. We sought to pilot a novel methodology using a self-sampling brush to obtain cervical cells for HPV DNA and RNA, RNA for vaginal cytokines, and cervical cytopathology to understand this disease process better and develop a means to make cervical monitoring more accessible and less costly to these high-risk, underserved women.

## Materials and Methods

### Study design

Thirty AA women with SLE recruited from the Rheumatology clinics consented to participate in the study. Inclusion criteria: AA women with SLE and meeting American College of Rheumatology (ACR), Systemic Lupus International Collaborating Clinics (SLICC), and 2019 European Alliance of Associations for Rheumatology (EULAR)/ACR classification criteria, ages 18–50 years, able to consent and obtain vaginal self-sampling.^7–10^ Exclusion criteria: current pregnancy, and not meeting inclusion criteria. Clinical information was obtained by records review and interviewing the patient, and surveys administered to assess cervical health information, knowledge of HPV, and satisfaction with using the self-sampling brush.

Vaginal self-sampling by the participant was performed after instruction and the brush immersed in an RNA-stable ThinPrep vial. Hologic ThinPrep automated processor was used for slide preparation and cytology was read by a board-certified cytopathologist. The vaginal self-sampling device (Evalyn Brush^®^) was provided by the manufacturer.^11^ There were no serious adverse events from the device in this study.

### HPV DNA and RNA

Cervical specimens were divided for separate DNA and RNA isolations. Genomic DNA was extracted using a standard lysis/Proteinase K/isopropanol precipitation method, followed by ethanol wash. RNA extraction was performed using the ROCHE High Pure Viral RNA Kit and frozen at −80°C for further analysis. HPV DNA detection and typing was carried out by competitive multiplex polymerase chain reaction (PCR) to amplify the E6 region of HPV, followed by probe-specific single base extension to discriminate between naturally occurring HPV present in the sample and the synthetic competitors included in the reaction. Matrix-assisted laser desorption/ionization time of flight mass spectroscopy allows separation of products on a matrix-loaded silicon chip array.^12^ For HPV RNA transcript analysis, reverse transcription was performed using the SuperScript IV VILO Kit after DNase digestion using pooled primers for the E6 regions of HPV genotypes detected in the DNA analysis (HPV16, HPV18, HPV31, HPV35, HPV39, HPV45, HPV51, HPV52, HPV56, HPV58, HPV59, HPV66, HPV68, HPV73, and HPV90).

### Cytokine RNA

The concentration of RNA extracted from the samples as detailed above was determined using the NanoDrop One spectrophotometer (Thermo Fisher, Waltham, MA) and purity of the RNA was assessed through the A260/A280 ratio. A custom-developed human cytokine 96-well panel assay (Bio-Rad Laboratories, Hercules, CA) was used to detect the mRNA for 12 cytokines (interleukin [IL]-2, tumor necrosis factor-a, interferon-g, IL-4, IL-5, IL-6, IL-10, IL-12A, IL-12B, IL-13, IL-1, and IL-17A) using the iTaq™ Universal SYBR^®^ Green One-Step RT-PCR Kit (Bio-Rad Laboratories, Hercules, CA) and run on the CFX96 qPCR machine (Bio-Rad).

Values were normalized to glyceraldehyde 3-phosphate dehydrogenase and calculated with 2^−ΔΔCt^ method as described.^13^ Assay data were then analyzed using the delta-delta Ct method with Bio-Rad CFX Manager 3.1 software. A heatmap was plotted using the ComplexHeatmap R package utilizing the ddCt values obtained from reverse transcription (RT)-PCR for the various cytokines. The 2^−ΔΔCt^ values for each measured cytokine was *Z*-score normalized across samples for the heatmaps.

For each cytokine, a positive *Z*-score for a sample indicates high expression in that sample while a negative *Z*-score indicates low expression in that sample. Additionally, the expression level was compared between the HPV-positive and the HPV-negative groups for each cytokine using the Mann–Whitney analysis. Boxplots were generated using the ggpubr package in R.

### Cervical cytology

For cervical cytology, the cells from the brush system were resuspended in 30 mL of RNA-stable ThinPrep provided by Hologic (ThinPrep System). The specimen was processed using the Hologic ThinPrep T2000 or T5000 automated Processor to prepare a microscope slide for cytopathological examination by a certified histotechnologist and board-certified cytopathologist. An interpretation of the smear was rendered using the Bethesda System for Reporting Cervical Cytology 3rd Edition.^14^

### Statistical analysis

Descriptive analysis was performed for the clinical features, survey results, and HPV DNA and RNA results. For the cytokine analyses, cytokine heat mapping was used with statistical analysis using machine learning to determine any correlations between cytokines and clinical and HPV variables using Student's *t*-test and nonparametric testing to determine any significance. Data for RT-PCR were generated by Bio-Rad CFX manager 3.1 software and the cytokine gene expression fold-change was calculated using the formula 2^−ΔΔCt^ and the Prism 9 Software (GraphPad, San Diego, CA). The heatmap was plotted using the ComplexHeatmap R package program and Boxplots were generated using the ggpubr package in R.

### Ethics

This study was approved by the Institutional Review Board (IRB) of Ascension St. John Hospital in Detroit, Michigan (IRB no. 16992642, www.irb.org).Written consent was obtained from each participant in the study.

## Results

### Clinical characteristics

All patients met established criteria for SLE with a mean age of 36.9 years (±9.4), and disease duration a mean of 14.5 years (±9.9). These women had moderate-to-severe disease, 40% of which had lupus nephritis and 16.7% neuropsychiatric lupus. All were treated with chronic immunosuppressives with 96.7% on corticosteroids, 96.7% on hydroxychloroquine, and 80% on nonsteroid immunosuppressives (Table 1).

**Table 1.** Patient Characteristics, *N* = 30

Patient characteristics	Mean or % (***n***/***N***)	SD	Range
#ACR **1997** criteria points met	6.9	±1.8	
#SLICC **2012** criteria points met	8.6	±2.1	
#EULAR/ACR **2019** criteria points met	23.5	±9.3	
Age (years)	36.9	±9.4	19–49
Years of SLE diagnosis	14.5	±9.9	1–36
Treatment			
HQ	96.7 (29/30)		
CS	96.7 (29/30)		
IS	80.0 (24/30)		
Nephritis	40.0 (12/30)		
CNS	16.7 (5/30)		

**Reproductive history**	**Mean or number of patients**	**SD**	**Range**
No. of pregnancies/patient	2.3	±2.5	0–11
No. of liveborn/patient	1.7	±1.4	0–5
No. of patients with stillbirths	2 (1 = HPV+, 1 = HPV−)		
No. of patients with miscarriages	12 (8 = HPV+, 4 = HPV−)		
No. of patients with voluntary interruption of pregnancy	9		
HPV detected	70.0 (21/30)		

**Insurance**	**% (*n*/*N*)**		
Medicare (disability)	30.0 (9/30)		
Medicaid	56.7 (17/30)		
Commercial	13.3 (4/30)		

ACR, American College of Rheumatology; CNS, central nervous system; CS, corticosteroids; EULAR, European Alliance of Associations for Rheumatology; HPV, human papilloma virus; HQ, hydroxychloroquine; IS, immunosuppressives; SD, standard deviation; SLE, systemic lupus erythematosus; SLICC, Systemic Lupus International Collaborating Clinics.

Most of the women had adverse cervical health (Table 2) and reproductive histories (Table 1). Two thirds had a history of abnormal pap smear ranging from atypical squamous cells of undetermined significance (ASCUS) to high-grade squamous intraepithelial lesion/cervical intraepithelial lesion 3 (CIN 3). Seven women previously had a cervical biopsy and six previously underwent a surgical procedure for abnormal cytology or genital warts. The average time to last pap smear was 3.2 years (±3.7 years, range 1–15 years) and varied widely.

**Table 2.** Gynecologic History, *N* = 30

History of abnormal pap smears and HPV	% (***n***/***N***) or mean	SD	range
Patients with abnormal pap smears (ASCUS to HSIL/CIN 3)	63.0 (19/30)		
Time to last pap smear (years)	3.2	±3.7	1–15
History of cervical biopsy	23.3 (7/30)		
History of surgery for abnormal pap smear or cervical biopsy	30.0 (9/30)		
Documentation of HPV testing	43.3 (13/30)		
Documented HPV + test	26.7 (8/30)		

**Factors for acquiring HPV infection**	**% (*n*/*N*) or mean**	**SD**	**range**
History of sexually transmitted disease other than HPV	70.0 (21/30)		
Vaccinated for HPV	16.7 (5/30)		
Age at first sexual intercourse (years)	16.5	±3.2	12–30
No. of lifetime sexual partners	9.5	±7	1–30
History of cigarette smoking	26.7 (8/30)		
Use of condoms	26.7 (8/30)		

ASCUS, atypical squamous cells of undetermined significance; CIN 3, cervical intraepithelial lesion 3; HSIL, high-grade squamous intraepithelial lesion.

Prior HPV test results were documented in the records in only 43.4%, of which eight were positive. Risk factors for acquiring HPV infection were prevalent in this cohort with history of sexually transmitted diseases other than HPV in 70%, not being vaccinated for HPV in 83.3%, multiple lifetime sex partners (mean = 9.5 ± 7), history of cigarette smoking (26.7%), early age of first sexual intercourse (mean = 16.5 years ±3.2 years), and not using condoms (73.3%). Pregnancy histories showed a mean of 2.3 pregnancies (±2.5) with a mean of 1.7 liveborn infants (±1.4).

Adverse pregnancy outcomes were prevalent in these women with 12 having history of miscarriages and 2 having stillbirths but there was no apparent association with sample positivity for HPV. Nine patients had voluntary interruption of pregnancies. Most of our patients had insurance that reflected poor socioeconomics or disability by insurance type. Of the 30 patients, 17 (56.7%) had Medicaid insurance, 9 (30%) had Medicare insurance (through disability), and only 4 (13.3%) had commercial insurance.

### Knowledge and attitudes

The women in general lacked knowledge of HPV-related oral and genital diseases other than cervical cancer (Table 3). Most of the women had knowledge that HPV can cause cervical cancer (70%), but were unaware that HPV can cause oral and other genital cancers (6.7% and 10%, respectively), head and neck cancer (6.7%), oral and genital warts (23.3% and 33.3%, respectively), and recurrent respiratory papillomatosis (6.7%). Regarding attitude toward the self-sampling device, most felt the device was easy to use (100%), comfortable (86.7%), and preferred it over traditional pap smear by gynecologic exam (80%).

**Table 3.** Knowledge and Attitudes, *N* = 30

	% (***n***/***N***)
Knowledge of HPV causing disease	
Cervical cancer	70.0 (21/30)
Genital warts	33.3 (10/30)
Oral warts	23.3 (7/30)
Recurrent respiratory papillomatosis	6.7 (2/30)
Anal cancer	13.3 (4/30)
Other genital cancers	10.0 (3/30)
Oral cancer	6.7 (2/30)
Head or neck cancer	6.7 (2/30)
Attitude toward using the self-sampling brush	
Easy/very easy to use	100 (30/30)
Comfortable/very comfortable	86.7 (26/30)
Preferred brush over traditional pap smears	80.0 (24/30)

### HPV DNA and RNA and cytology

Most of the samples were positive for HPV DNA 73.3% (21/30) with about half of these (10/21 samples) being positive for multiple types (Table 4). This is increased above the reported prevalence of high-risk genital HPV of 20.4% for all women and 28.2% for non-Hispanic AA women 18–69 years of age in the US.^15^ All HPV-positive samples showed active infection (+RNA). Most of the types identified were the well-described high-risk types and some newly identified as potential high-risk types, 73 and 90. HPV 16 and 18 were identified in only eight samples (26.7%), with 3 showing type 18, 6 showing type 16, with one sample containing both 16 and 18 types. Only five women were vaccinated for HPV, and of those, three were positive for HPV, including types not covered by the bivalent and quadrivalent vaccines: one tested positive for HPV 52, one for HPV 90, and one for both HPV 31 and 56, types not included in the bivalent or quadrivalent vaccines, but neither had HPV 16 or HPV 18 that are included in both vaccines.

**Table 4.** Human Papilloma Virus DNA and RNA and Cytology Results, *N* = 30

Sample ID	DNA HPV PCR-MA result	RNA multiplex HPV result	Cytology	Vaccinated for HPV	Other findings on cytology
W10	HPV35	Positive	N		BV
W11	HPV16 HPV18 HPV35 HPV59	Positive	LSIL		
W12	HPV31 HPV52 HPV56 HPV73 HPV90	Positive	N		
W14	HPV39 HPV66 HPV68	Positive	N		BV, fungi
W17	HPV52	Positive	N	YES	Parakeratosis
W18	HPV18	Positive	N		BV
W19	HPV90	Positive	N	YES	Trichomonas
W2	HPV45 HPV52 HPV59 HPV66	Positive	N		
W20	HPV45	Positive	N		
W21	HPV31	Positive	N		BV
W23	HPV18 HPV58	Positive	N		Hyperkeratosis
W25	HPV16 HPV31 HPV90	Positive	N		Hyperkeratosis
W27	HPV31 HPV56	Positive	N	YES	
W28	HPV31 HPV51	Positive	ASCUS		
W29	HPV16	Positive	N		BV
W3	HPV16	Positive	N		
W4	HPV16	Positive	N		BV
W6	HPV39	Positive	N		Fungi-Candida
W7	HPV31 HPV35 HPV51 HPV56	Positive	LSIL		Shift in vaginal flora suggesting vaginosis
W8	HPV16 HPV90	Positive	LSIL		
W9	HPV51	Positive	N		
W1	Negative	NA	N		
W5	Negative	NA	N	YES	
W13	Negative	NA	N		Fungi
W15	Negative	NA	N		BV
W16	Negative	NA	N		
W22	Negative	NA	N	YES	BV, keratotic
W24	Negative	NA	N		BV
W26	Negative	NA	N		
W30	Negative	NA	N		

BV, bacterial vaginosis; LSIL, low-grade squamous intraepithelial lesion; MA, mass spectroscopy; NA, not applicable; PCR, polymerase chain reaction.

The nonavalent vaccine was implemented in 2016, which was after the women in our study would have been eligible to receive this vaccine. Of note, of the vaccinated women showing +HPV, only HPV 90 is not included in the nonavalent HPV vaccine.

Cytology was performed on all samples using standard techniques applied to samples in the outpatient setting as detailed under methods. Cell samples obtained from the brush showed preservation of morphology, but quality indicators were generally absent. Abnormal cytology was detected in 13.3% (4/30) with three showing low-grade squamous intraepithelial lesion and one showing ASCUS. This is above the expected population rate of 5.1%.^16^ All four samples with abnormal cytology were positive for multiple HPV types. In addition, 53% (16/30) showed other non-neoplastic abnormalities on cytology, such as bacterial vaginosis, trichomonas, parakeratosis, keratosis, and presence of fungi. All women were notified of the research study cytology findings and HPV testing and strongly advised to see their gynecologist for a gynecological exam with a pap smear and HPV testing. Most patients were not aware when notified that they had an HPV infection.

### Cytokine RNA analyses

**FIG. 1.**
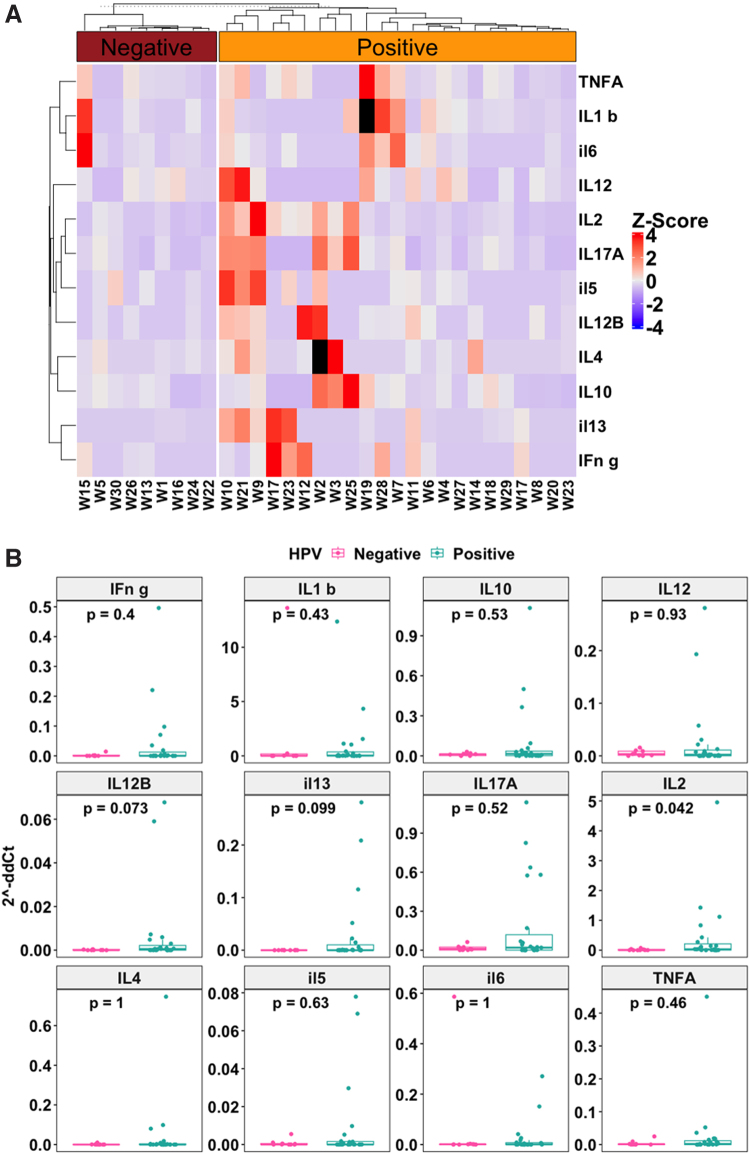
**(A)** Cytokine heatmap. High expression of tested cytokines was associated with HPV positivity. Heatmap of *Z*-score normalized cytokine 2^−ddCt^ values across samples. The *Z*-score scale ranges from low expression (blue) to high expression (red) for each cytokine shown. Two samples, W19 and W2, had missing values for IL-1b and IL-4, respectively, and so had no *Z*-score values for those cytokines on the heatmap. **(B)** Cytokine BoxPlots. High expression of tested cytokines was associated with HPV positivity. Boxplots comparing expression levels between HPV-negative samples and HPV-positive samples for each cytokine. *p*-Values shown were computed using Mann–Whitney test. *n* = 9 for HPV-negative samples and *n* = 21 for HPV-positive samples. HPV, human papilloma virus; IL, interleukin.

The cytokine RNA analyses showed that HPV-negative samples had low gene expression for all cytokines tested (Fig. 1A, B). In contrast, upregulation of cytokine RNA was noted mainly in the HPV-positive samples compared with HPV-negative samples (Fig. 1A). Only one HPV-negative sample exhibited increased level of cytokine expression. Similarly, the Mann–Whitney analyses showed higher levels of cytokine expression in HPV-positive samples compared with HPV-negative samples (Fig. 1B). Only IL-2 showed statistically significant higher expression in HPV-positive samples compared with HPV-negative samples (*p* = 0.042). Two cytokines, IL-12B (*p* = 0.073) and IL-13 (*p* = 0.099), also showed a trend of higher expression in HPV-positive samples compared with HPV-negative samples. However, these were not statistically significant at *p* = 0.05 significance threshold.

## Discussion

To our knowledge, this is the first study to assess HPV DNA and RNA and vaginal RNA cytokine profiles in AA women with SLE, who are high risk for HPV infection and cervical cancer. In addition, we used a novel self-sampling technique to collect cervical cells and showed that cell morphology is preserved, and cytology can be determined using the standard technique for processing and reading pap smears in the outpatient setting. HPV infection is a serious health issue for women with SLE as they have a higher incidence of genital infection and cervical neoplasia.^1–3^ The immune suppression in SLE likely creates a facilitative environment for persistence of HPV infection and subsequent malignant transformation. Just having SLE as a diagnosis was shown to be associated with increased prevalence of premalignant lesions even though these patients had fewer classical risk factors. SLE patients also are exposed to long-term immunosuppression, which has been shown to increase the chance of presenting with more premalignant lesions.^17^

Cervical cancer screening remains problematic for these women as current screening guidelines are inadequate for these women. Current guidelines recommend initial screening at age 21 years, then every 3 years from age 21–29 without HPV cotesting. For ages 30–65, screening either every 5 years along with HPV cotesting, cytology alone every 3 years, or FDA-approved high-risk HPV testing alone every 5 years.^18,19^

For the HIV population, who are considered at increased risk for cervical cancer, pap smear screening is recommended in the first year following diagnosis of HIV (regardless of age), then yearly thereafter for 3 years. After that, if three consecutive pap smears are normal, then pap smear testing can be done every 3 years. HPV testing should be done following the diagnosis of HIV. If HPV cotesting is available, then cotesting can be done at the time of diagnosis of HIV, and if positive for HPV 16 or 18, then colposcopy should be performed.^20^ There are no specific screening guidelines for women with SLE who are immunosuppressed similarly to those with HIV.

Although most of the samples lacked quality indicators for cytology, we did detect abnormal findings in 13.3%, which is more than twice the expected population rate of 5.6%.^16^ Thus, although the cytology using the brush sampling may be less sensitive than the traditional pap smear, a less sensitive test done more frequently is better than no testing.

Newly acquired HPV infections generally clear or become undetectable in 90% within 2 years, which was previously thought to signal viral clearance from the cervix.^21,22^ In women with SLE followed for 3 years, about half (48.5%) of incident infections persisted ≥6 months and only 14.7% of these were cleared during the study period.^23^ More studies now support the concept of HPV latency with HPV remaining dormant expressing a low copy number in infected stem cells of the basal cervical cell layer with intermittent reactivation.^24–28^

Local cell-mediated immunity maintains immune control of HPV infection. Continuous surveillance by resident T memory cells in the cervical epithelium downregulates HPV gene expression and keeps the HPV dormant.^24^ Reactivation of dormant HPV infections can occur in situations where host immune surveillance in the cervical microenvironment is perturbed, such as with aging, prolonged contraception, smoking, or immunosuppression.^29–31^ Increased redetection of HPV after clearance has been reported in women with HIV, organ transplant recipients, and patients on immunosuppressive drugs.^32,33^ In addition, women who are abstinent show redetection of HPV.^31,34^

HPV has been shown to be latent and not actively being transcribed in dysplastic/neoplastic cervical tissue in women with SLE.^35^ Although natural infection does not generally result in protective antibodies, HPV vaccination boosts humoral immunity and may protect against reactivation of latent infection.^36^ Our cohort showed that the majority of women had active HPV infections that they were not aware of and half of them were infected with multiple HPV types. In addition, risk factors for acquiring HPV infections were prevalent in our cohort. The technique we used to detect HPV DNA and RNA is very sensitive and can detect small quantities of HPV DNA, which is not normally detected using commercial testing and would be missed in the normal outpatient setting.

The role of cytokines in the microenvironment of the cervix is an evolving area of study. The cytokine microenvironment may promote latency, persistence, and progression to cervical cancer. Cytokines in the cervix are predominantly secreted by CD4^+^ T lymphocytes and macrophages, which maintain local immune surveillance and cell-mediated immunity. Studies suggest that the HPV virus may alter the local cytokine environment to suppress the immune system and evade immune surveillance.^37,38^ In addition, aging and immune suppression may result in cytokine microenvironment changes that facilitate reactivation of latent infection or persistence and neoplastic transformation.^39,40^

HPV infection is associated with increased expression of proinflammatory cytokines as well as upregulation of immunosuppressive cytokines and regulatory T cells.^39,41^ It is postulated that a decrease in T helper type 1 response or cell-mediated immunity and an increase in T helper type 2 response or humoral immunity is associated with neoplastic transformation, but studies are not consistent^42–44^ with one study showing increased intralesional interferon gamma to be associated with oncogenic potential.^45^

We showed a trend of cytokine up in HPV-positive samples. Only expression of IL-2 was statistically significant in HPV-positive samples compared with HPV-negative samples. For the rest of the cytokines, although there were shown associations with HPV infection, they were not statistically significant, likely due to the small sample size.

The limitations of our study were that the sample size was small, and we could not achieve normal distribution of data for statistical purposes. In addition, most of the cytology specimens lacked quality indicators so would be less sensitive to detect cytologic abnormalities, which were likely underestimated in this study. In addition, our HPV DNA and RNA testing in this research setting was much more sensitive than the commercial Clinical Laboratory Improvement Amendments (CLIA)-approved laboratory testing done in the outpatient setting so we could not officially report the results to the medical record.

Cervical cancer is a preventable disease and risk can be reduced by simple measures such as condom use and HPV vaccination. Awareness, education, and access to testing are key to engage high-risk women to reduce their cervical cancer risk. The women in our study were not aware of the spectrum of HPV-related diseases and were not using simple health prevention measures to protect themselves. HPV vaccination rates in AA adults are 10% lower than Caucasians (45.2% vs. 56.5%, respectively) but in our cohort, the rate was much lower at 16.7% indicating that being a woman of color is not the sole determinant of poor vaccine uptake.^46^

In view of mounting evidence that HPV infections become latent rather than clear, particularly in immunosuppressed populations, preventing infection remains the best option for addressing the increased rates of HPV-related disease and cervical cancer in AA women with SLE. In addition to prevention of infection, HPV vaccination has been associated with lower redetection rates in previously infected women indicating that the vaccine may function to maintain the dormancy of HPV.^36^ The quadrivalent HPV vaccine has been shown to be safe and immunogenic in women with SLE.^47^

HPV vaccine uptake is low in women with SLE, however, despite the increased risk for HPV infection in this population.^48^

## Conclusion

In conclusion, risk factors for HPV infection were prevalent in our cohort, yet these women were not engaging in these simple prevention strategies. Primary care doctors are likely unaware of this increased risk of HPV infection and cervical neoplasia in AA women with SLE, and thus may not be advocating for close cervical monitoring and HPV vaccination in these patients. AA women with SLE are also likely unaware of their increased risk for HPV infection and cervical neoplasia and not advocating for themselves to request more frequent testing, nor adhering to regular pap smear and HPV testing with the current guidelines. Awareness, education, and access to testing are key to engage high-risk women to reduce their cervical cancer risk. The self-sampling brush has the potential to make cancer screening more accessible as well as allow more frequent cervical monitoring at lower cost. This also raises the question as to whether screening should be done earlier than 21 years of age for those who are sexually active and on aggressive immunosuppression.

We have just touched the surface of this problem, which needs further studies to better delineate the biology of HPV infection in AA women with SLE. However, it is clear that we need educate and empower these high-risk women to advocate for themselves and take control of their cervical health.

## Abbreviations Used

AA: African American
ACOG: American College of Obstetricians and Gynecologists
ACR: American College of Rheumatology
ASCUS: atypical squamous cells of undetermined significance
BV: bacterial vaginosis
CIN 3: cervical intraepithelial lesion 3
CLIA: Clinical Laboratory Improvement Amendments
CNS: central nervous system
CS: corticosteroids
EULAR: European Alliance of Associations for Rheumatology
HPV: human papilloma virus
HQ: hydroxychloroquine
HSIL: high-grade squamous intraepithelial lesion
IL: interleukin
IRB: Institutional Review Board
IS: immunosuppressives
LSIL: low-grade squamous intraepithelial lesion
MA: mass spectroscopy
NA: not applicable
RT-PCR: reverse transcription-polymerase chain reaction
SD: standard deviation
SLE: systemic lupus erythematosus
SLICC: Systemic Lupus International Collaborating Clinics
USPSTF: United States Preventive Services Task Force

## References

[1] Dhar JP, Kmak D, Bhan R, et al. Abnormal cervicovaginal cytopathology in women with lupus: A retrospective cohort study. Gynecol Oncol 2001;82:4–6. 11426953 10.1006/gyno.2001.6207

[2] Dhar JP, Essenmacher L, Ager J, et al. Ominous cervical cytopathology in women with lupus. Int J Gynecol Obstet 2005;89:795–796. 10.1016/j.ijgo.2005.02.006 15919405

[3] Tam LS, Chan AYK, Chan PKS, et al. Increased prevalence of squamous intraepithelial lesions in systemic lupus erythematosus. Association with human papilloma virus. Arthritis Rheum 2004;50(11):3619–3625. 10.1002/art.20616 15529372

[4] Bernard V, Watson M, Saraiya M, et al. Cervical cancer survival in the United States by race and stage (2001–2009): Findings from CONCORD-2 study. Cancer 2017;123(Supplement 24):5178–5189. 29205300 10.1002/cncr.30906 PMC6386458

[5] Singh GK, Jemal A. Socioeconomic and racial/ethnic disparities in cancer mortality, incidence, and survival in the United States, 1950–2014: Over six decades of changing patterns and widening inequalities. JEnviron Public Health 2017;2017:2819372; doi: 10.1155/2017/2819372 28408935 PMC5376950

[6] Demas K, Costenbader K. Current opinion in rheumatology review. Curr Opin Rheumatol 2009;21(2):102–109. 19339919 10.1097/BOR.0b013e328323daad PMC2774141

[7] Tan EM, Cohen AS, Fries JF, et al. The 1982 revised criteria for the classification of systemic lupus erythematosus. Arthritis Rheum 1982;25(11):1271–1277; doi: 10.1002/art.1780251101 7138600

[8] Hochberg MC. Updating the American College of Rheumatology revised criteria for the classification of systemic lupus erythematosus. Arthritis Rheum 1997;40(9):1725; doi: 10.1002/art.1780400928 9324032

[9] Petri M, Orbai AM, Alarcón GS, et al. Derivation and validation of the Systemic Lupus International Collaborating Clinics classification criteria for systemic lupus erythematosus. Arthritis Rheum 2012;64(8):2677–2686; doi: 10.1002/art.34473 22553077 PMC3409311

[10] Aringer M, Costenbader K, Daikh D, et al. 2019 European League Against Rheumatism/American College of Rheumatology classification criteria for systemic lupus erythematosus. Arthritis Rheumatol 2019;71(9):1400–1412; doi: 10.1002/art.40930 31385462 PMC6827566

[11] Available from: https://www.roversmedical devices.com/cell-sampling-devices/evalyn-brush [Last accessed: April 12, 2023].

[12] Walline HM, Komarck C, McHugh JB, et al. High risk human papillomavirus detection in oropharyngeal, nasopharyngeal, and oral cavity cancers: Comparison of multiple methods. JAMA Otolaryngol Head Neck Surg 2013;139(12):1320–1327; doi: 10.1001/jamaoto2013.5460 24177760 PMC4049419

[13] Livak KJ, Schmittgen TD. Analysis of relative gene expression data using real-time quantitative PCR and the 2^-ΔΔCT^ method. Methods 2001;25:402–408. 11846609 10.1006/meth.2001.1262

[14] The Bethesda System for Reporting Cervical Cytology, 3rd ed. (Nayar R, Wilbur D. eds.) Springer-Verlag: New York, NY, USA; 2014.

[15] McQuillan G, Kruszon-Moran D, Markowitz LE, et al. The Prevalence of HPV in Adults Aged 18–69: United States, 2011–2014. NCHS Data Brief, No 280. National Center for Health Statistics: Hyattsville, MD, USA; 2017.

[16] Sadeghi, S, Sadeghi A, Robboy S. Prevalence of dysplasia and cancer of the cervix in a nationwide, planned parenthood population. Cancer 1988;61:2359–2361. 3365664 10.1002/1097-0142(19880601)61:11<2359::aid-cncr2820611135>3.0.co;2-7

[17] Klumb, EM, Araujo ML, Jesus GR, et al. Is higher prevalence of cervical intraepithelial neoplasia in women with lupus due to immunosuppression? J Clin Rheumatol 2010;16(4):153–157. 20407390 10.1097/RHU.0b013e3181df5261

[18] Updated Cervical Cancer Screening Guidelines. Practice Advisory April 2021. The American College of Obstetricians and Gynecologists. Available from: https://www.acog.org/clinical/clinical-guidance/practice-bulletin [Last accessed: April 12, 2023].

[19] USPSTF Final Recommendations Statement Cervical Cancer Screening. August 21, 2018. Available from: www.uspreventitive servicestaskforce.org/ [Last accessed: April 12, 2023].

[20] Guidelines for the Prevention and Treatment of Opportunistic Infections in Adults and Adolescents with HIV. National Institutes of Health, Centers for Disease Control and Prevention, HIV Medicine Association, and Infectious Diseases Society of America; Q1–Q30. Available from: https://clinicalinfo.hiv.gov/ed/guidelines/adult-and-adolescent-opportunistic-infection [Last accessed: January 21, 2023].

[21] Satterwhite C, Torrone E, Meites E, et al. Sexually transmitted infections among US women and men: Prevalence and incidence estimates 2008. Sex Transm Dis 2013;40(3):187–193. 23403598 10.1097/OLQ.0b013e318286bb53

[22] Skinner R, Wheeler C, Romanowski B, et al. for the VIVIANE study group. Progression of HPV infection to detectable cervical lesions or clearance in adult women: Analysis of the control arm of the VIVIANE study. Int J Cancer 2016;138:2428–2438. 26685704 10.1002/ijc.29971 PMC4787275

[23] Tam L, Chan PKS, Ho S, et al. Natural history of cervical papilloma virus infection in systemic lupus erythematosus-a prospective cohort study. J Rheumatol 2010;37(2):330–340. 20032093 10.3899/jrheum.090644

[24] Gravitt P. The known unknowns of HPV natural history. J Clin Invest 2011;121(12):4593–4599. 22133884 10.1172/JCI57149 PMC3225991

[25] Doorbar J. Latent Papillomavirus infections and their regulation. Curr Opin Virol 2013;3:416–421. 23816390 10.1016/j.coviro.2013.06.003

[26] Hammer A, Koning M, BL aakaer J, et al. Whole tissue cervical mapping of HPV infection: Molecular evidence for focal latent HPV infection in humans. Papillomavirus Res 2019;7:82–87. 30772498 10.1016/j.pvr.2019.02.004 PMC6389775

[27] Ermel A, Shew M, Imburgia T, et al. Redetection of human papillomavirus type 16 infections of the cervix in mid-adult life. Papillomavirus Res 2018;8:75–79. 10.1016/j.pvr.2018.01.001 PMC5886910 29355777

[28] Gravitt P. Evidence and impact of human papillomavirus latency. Open Virol J 2012;6 (supple 2:M5):198–203. 23341855 10.2174/1874357901206010198 PMC3547385

[29] Gaducci A, Barsotti C, Cosio S, et al. Smoking habit, immune suppression, oral contraceptive use and hormone replacement therapy us and cervical carcinogenesis: A review of the literature. Gynecol Endocrinol 2011;27:597–604. 21438669 10.3109/09513590.2011.558953

[30] Shew M, Ermel A, Tong Y, et al. Episodic detection of human papillomavirus within a longitudinal cohort of young women. J Med Virol 2015:87:2122–2129. 26112742 10.1002/jmv.24284

[31] Rositch A, Burke A, Viscidi R, et al. Contributions of recent and past sexual partnerships on incident human papillomavirus detection: Acquisition and reactivation in older women. Cancer Res 2012:72(23):6183–6190. 23019223 10.1158/0008-5472.CAN-12-2635 PMC3513486

[32] Theiler R, Farr S, Karon J, et al. High-risk human papillomavirus reactivation in human immunodeficiency virus-infected women. Risk factors for viral shedding. Obstetr Gynecol 2010;115(6):1150–1158. 10.1097/AOG.0b013e3181e00927 20502284

[33] Hinten F, Hilbrands L, Meeuwis K, et al. Reactivation of latent HPV infections after renal transplantation. Am J Transplant 2017;17:1563–1573. 28009475 10.1111/ajt.14181

[34] Fu TC, Carter JJ, Hughes JP, et al. Re-detection vs. new acquisition of high-risk human papillomavirus in mid-adult women. Int J Cancer 2016;139:2201–2212. 10.1002/ijc.30283 PMC5222584 27448488

[35] Dhar JP, Gregoire L, Lancaster WD, et al. Evaluation of high-risk HPV (HPV-16 and -18) RNA and integration in cervical neoplasms in systemic lupus erythematosus. Curr Womens Health Rev 2013;9(3):189–193.

[36] Wheeler CM, Skinner SR, Del Rosario-Raymundo M, et al. Efficacy, safety and immunogenicity of the human papillomavirus 16/18 ASO4-adjuvanted vaccine in women older than 25 years: 7-year Follow-up of the Phase 3, Double-blind, Randomized, Controlled Viviane Study. Lancet Infect Dis 2016;16:1154–1168. 27373900 10.1016/S1473-3099(16)30120-7

[37] Bonin C, Padovani C, da Costa I, et al. Detection of regulatory T cell phenotypic markers and cytokines in patients with human papillomavirus infection. J Med Virol 2019;91:317–325. 30192406 10.1002/jmv.25312

[38] Nicol A, Nuovo G, Wang Y, et al. In situ detection of SOCS and cytokine expression in the uterine cervix from HIV/HPV co-infected women. Exp Mol Pathol 2006;81:4–47. 10.1016/j.yexmp.2006.01.002 16878360

[39] Marks M, Viscidi R, Chang K, et al. Differences in the concentration and correlation of cervical immune markers among HPV positive and negative perimenopausal women. Cytokine 2011;56(3); doi: 10.1016/j.cyto.2011.09.012 PMC3831521 22015106

[40] Hwang L, Scott M, Ma Y, et al. Higher levels of cervicovaginal inflammatory and regulatory cytokines and chemokines in healthy young women with immature cervical epithelium. J Reprod Immunol 2011;88:66–71. 21051089 10.1016/j.jri.2010.07.008 PMC3049722

[41] Nicol A, Fernandes A, Grinsztejn B, et al. Distribution of immune cell subsets and cytokine-producing cells in the uterine cervix of human papillomavirus (HPV)-infected women. Influence of HIV coinfection. Diagn Mol Pathol 2005;14(1):39–47. 15714063 10.1097/01.pas.0000143309.81183.6c

[42] Azar K, Tani M, Yasuda H, et al. Increased secretion patterns of interleukin 10 and tumor necrosis factor -alpha in cervical squamous intraepithelial lesions. Human Pathol 2004;35(11):1376–1384. 15668895 10.1016/j.humpath.2004.08.012

[43] Daniilidis A, Koutsos J, Oikonomou Z, et al. Cytokines of cervical mucosa and human papilloma virus infection of the cervix: A descriptive study. Gynecol Cytopathol 2016;60:58–64. 10.1159/000445161 27003414

[44] Iwata T, Fujii T, Morii K, et al. Cytokine profile in cervical mucosa of Japanese patients with cervical intra-epithelial neoplasia. Int J Clin Oncol 2015;20:126–133. 24578180 10.1007/s10147-014-0680-8

[45] Song S, Lee J, Seok O, et al. The relationship between cytokines and HPV-16, HPV 16 E6, E7 and high-risk HPV viral load in the uterine cervix. Gynecol Oncol 2007;104:732–738. 17188341 10.1016/j.ygyno.2006.10.054

[46] CDC 2022. Vaccination Coverage among Adults in the United States. Estimated proportion of adults aged 19–26 years who received at least one dose of human papillomavirus (HPV) vaccine by age group, sex, and race/ethnicity. National Health Interview Survey, United States, 2018. Suppl. Box 4. Available from: https://www.cdc.gov/mmwr/volumes/79/ss/ss7003a1.htm

[47] Dhar JP, Essenmacher L, Dhar R, et al. The safety and immunogenicity of quadrivalent HPV (qHPV) vaccine in systemic lupus erythematosus. Vaccine 2017;35(20):2642–2646. 28404357 10.1016/j.vaccine.2017.04.001

[48] Dhar JP, Essenmacher L, Dhar R, et al. Lack of uptake of prophylactic human papilloma virus vaccine among women with systemic lupus erythematosus seen at a Regional Medical Center. J Clin Rheumatol 2019;25(8):348–350; doi: 10.1097/RHU.0000000000000866 31764496

